# A case series of upper extremity reconstructions utilizing partial ECRB and ECRL tendon autografts

**DOI:** 10.1093/jscr/rjae278

**Published:** 2024-08-14

**Authors:** David Haddad, Darren Kempton, Joey Ghotmi, Tolga Türker

**Affiliations:** Department of Orthopaedic Surgery, The University of Arizona College of Medicine-Tucson, 1501 N. Campbell Ave., Floor 8, Tucson 85719, AZ, United States; Department of Dermatology, The University of New Mexico School of Medicine, 915 Camino de Salud, NE Albuquerque, NM 87106, United States; The University of Arizona College of Medicine-Tucson, 1501 N. Campbell Ave., Floor 8, Tucson 85719, AZ, United States; Department of Orthopaedic Surgery, The University of Arizona College of Medicine-Tucson, 1501 N. Campbell Ave., Floor 8, Tucson 85719, AZ, United States

**Keywords:** autograft, ECRB, ECRL, harvest, tendon

## Abstract

Sources of autografts such as palmaris longus or plantaris are often limited or absent. We present our experience using a low donor-site morbidity method of harvesting strips of extensor carpi radialis brevis and longus (ECRB and ECRL) as free tendon autografts in upper extremity soft tissue reconstructions. Retrospective chart review identified five patients who received reconstructive upper extremity surgeries using ECRB and ECRL partial tendon autografts from January 2014 to October 2021 with at least a 12-month follow-up period. Mayo wrist scores were calculated to demonstrate clinical outcomes. All five patients (mean follow-up: 21 months) were able to return to regular activities while demonstrating improvements in 6- and 12-month postoperative Mayo wrist scores. There was minimal donor site morbidity and no ruptures of parent tendons following harvest. This study provides additional support for utilizing partial strips of ECRB and ECRL in repairing upper extremity tendon gap and ligament deformities.

## Introduction

Complex extremity injuries often present as challenging surgical conditions which may require utilization of tendon grafts [1, 2]. Several different sources for these grafts have been well described to accomplish these surgeries [2]. Allografts reduce the risk of donor-site morbidity and are readily available, but carry risks of slow incorporation, controversial strength profiles, and high costs due to commercial sterilization [3, 4]. Autografts are biomechanically strong and inherently incorporate well into the recipient site [5]. However, the harvesting process can result in local neuromas, scarring, pain, tendonitis, or rupture [6]. In addition, if a severe injury requires a considerable amount of autograft tendons, alternative sources may be limited or absent. This can be seen with palmaris longus (PL) and plantaris, which are frequently found to have variable anatomy and insufficient for grafting nearly 60% of the time [7, 8].

Recent literature has sought to contribute a low donor-site morbidity method of harvesting strips of extensor carpi radialis longus (ECRL) and extensor carpi radialis brevis (ECRB) as free tendon autografts [9]. Biomechanical data has shown that wrist motion is preserved post-partial harvest due to compensation from surrounding muscles, such as extensor carpi ulnaris [10–12]. Additionally, the majority of upper extremity reconstructions using strips of ECRB and ECRL tendons are distally based and limited to local structures by pedicles [13–19]. However, there is a paucity of research regarding procedures in which these tendons are freed from their base attachments to salvage reconstructions for patients with few alternatives. The objective of this study is to present our experience using these partial tendon autografts in our clinical practice. We believe that this study may help in further promoting this technique to surgeons who are seeking reliable sources of autograft tendons to use in their patients in which other options are exhausted or not available due to injury.

## Materials and methods

### Statement of ethics

This study is a hospital and Institutional Review Board approved retrospective chart review of patients who received reconstructive surgeries using ECRL and ECRB partial autografts in our practice from January 2014 to October 2021. Formal review and approval were given by the BLINDED Institutional Review Board. All patient information was de-identified which allowed for consent to be waived.

### Inclusion and exclusion criteria

We included patients of all age groups who had extensive tendon or ligament gaps for which they required a tendon graft for salvage reconstruction. Contraindications included patients with scarring of the dorsal forearm and/or to the ECRL and ECRB tendons, collagen disorders, acute or chronic forearm infections, or neurological conditions. These patients were not eligible for this technique and were given other tendon graft options to accomplish reconstruction. The technique used for all surgeries was followed as previously described in the literature [9].

### Collected patient variables

All data obtained from reviewing patient charts was stored in a password protected and encrypted Health Insurance Portability and Accountability Act compliant platform. Our data consisted of demographics (age, sex, tobacco use, musculoskeletal relevant co-morbidities), details of the injury (left or right side, level in extremity, type, mechanism of action, affected tendons, and associated structures), length of harvested tendon (equal to the gap requiring reconstruction), and treatment (prior procedures, time from injury to surgery, side of harvest, tourniquet time/pressure, and amount of blood loss).

### Outcome measures

Outcomes were reported as Mayo wrist scores due to concerns of utilizing muscles which act as centralizing forces in the distal upper extremity [12, 20]. Postoperative wound care was standardized as consolidating the same dressing and splint for tendon harvest and operative sites when in the same limb and separate when in different extremities. Each patient was referred to a certified occupational hand therapist and treatments were tailored according to the type of reconstructions.

## Results

**Figure 1:**
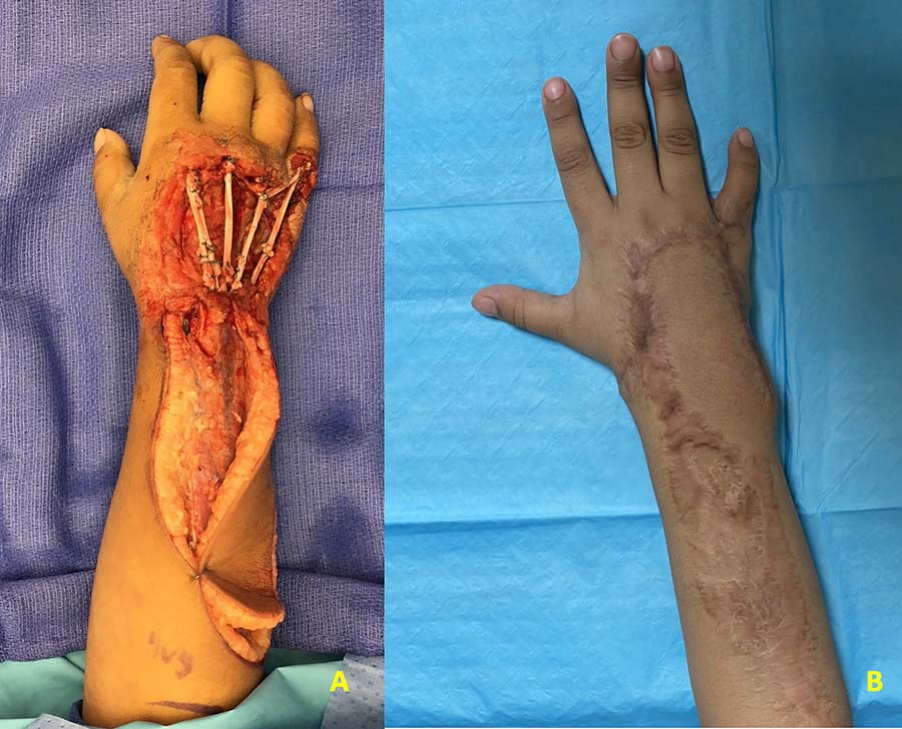
(A) Patient 4 intraoperative photograph of extensor tendon reconstruction using autografts. (B) Patient 4 postoperative photograph at 6 months.

**Figure 2:**
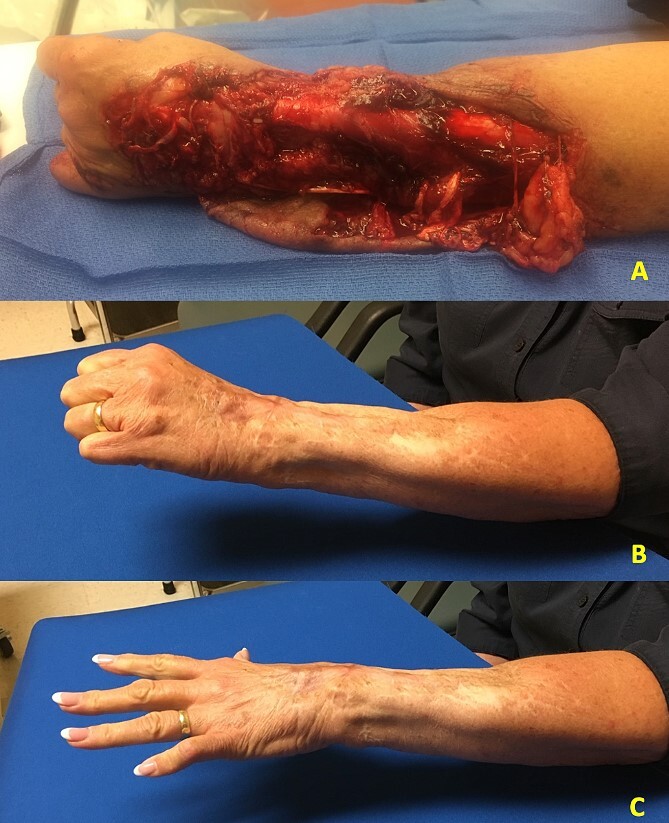
(A) Patient 1 preoperative photograph showing laceration and degloving injury to the forearm with loss extensor tendons. (B and C) Patient 1 postoperative photographs showing functional results at 12 months.

**Figure 3:**
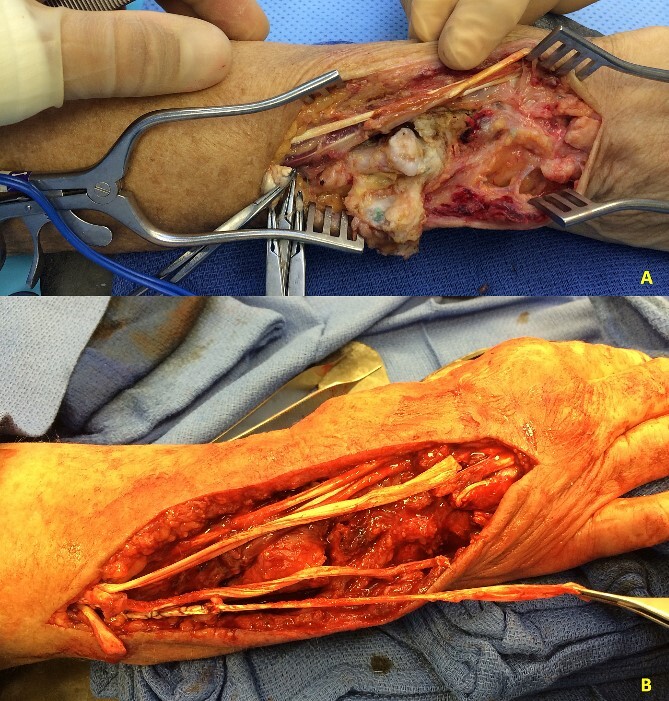
(A) Patient 5 intraoperative photograph showing chronic caput ulnae syndrome. (B) Patient 5 intraoperative extensor tendon reconstruction using autografts.

There were five patients identified in our review of medical records. The mean age was 44.8 years old (range: 5–69) and the median was 41 (Table 1). A description of injuries can be found in Table 2. The mean time to surgery was 247 days (range: 2–949) and the median was 36.4 days. This can be found along with treatment details in Table 3. Patients 1–5 all had at least a 12-month follow up, with a mean follow-up time of 21 months (Figs 1–3). Two patients were excluded from the functional score section due to not meeting at least a 6-month requirement of clinical information. All five patients otherwise were able to return to work and showed improved Mayo wrist scores at both 6- and 12-months (Table 4).

**Table 1:** Patient demographics.

**Patient**	**Age (years)**	**Sex**	**MSK Co-morbidities**	**Tobacco use**
1	69	F	None	No
2	41	M	None	No
3	40	M	None	Yes
4	5	M	None	No
5	69	F	AS, Osteopenia	No

Notes: MSK, Musculoskeletal. F, Female. M, Male. AS, Ankylosing Spondylitis.

**Table 2:** Primary and associated injuries.

**Patient**	**Injury side**	**Injury Level**	**Mechanism of Injury**	**Type of injury**	**Injured structure and graft size for defect**	**Associated ipsilateral injuries**
1	L	Forearm	MVC	Laceration Degloving	EPL (4 cm), EIP (3 cm), EDC index (3 cm), long (3 cm), and ring fingers (2.5 cm)	Ulnar fracture (comminuted, open)
2	L	Hand	MVC	Crush Degloving	EDC (16 cm), long (16 cm), ring (16 cm), and small fingers (16 cm), EIP (not repaired)	Index finger metacarpal base fracture (displaced, open), Extensive soft tissue loss
3	L	Forearm, Elbow	MVC	Degloving	EPL (14 cm), EDC index, long, ring, small fingers (14 cm per EDC)	Elbow dislocation (Open), Elbow and wrist arthrotomies (Open)
4	R	Hand	MVC	Crush	EIP (3 cm), EDC index (3 cm), long (3 cm), ring (3 cm), and small fingers (2 cm), EDM (2.5 cm)	Small finger proximal phalanx fracture (nondisplaced**,** open), Soft tissue defect dorsal hand, ring and small finger MCP arthrotomies
5	R	Hand	Caput ulnae syndrome	Rupture	EDC long (12 cm), ring (12 cm), and small fingers (12 cm)	DRUJ arthritis and capsule injury, Scapholunate ligament injury, TFCC tear, Thumb CMC joint osteoarthritis

Notes: L, Left, R, Right. Cm, Centimeters. MVC, Motor Vehicle Collision. EPL, Extensor Pollicis Longus. EDC, Extensor Digitorum Communis. EIP, Extensor Indicis Proprius. EDM, Extensor Digitorum Minimi. DRUJ, Distal Radioulnar Joint. CMC, Carpometacarpal. MCP, Metacarpophalangeal. TFCC, Triangular fibrocartilage complex.

**Table 3:** Descriptions of prior and end treatments.

**Patient**	**Treatment prior to tendon repair (time to reconstruction)**	**Time to reconstruction**	**Procedure**	**Side/% ECRB graft harvest**	**Side/% ECRL graft harvest**	**Tourniquet time/pressure**	**Blood loss**
1	Wound VAC application, I&D, Closed reduction of ulna fracture (All 2 days)	2 days	ECRL and ECRB harvest for reconstruction of EDC for index, long and ring fingers, EPL, and EIP, Full-thickness skin grafting to forearm, ORPF of ulnar fracture	R/50%	R/50%	60 min/250 mm Hg	<10 mL
2	Extensive I & D (6 months), Extensor tendon rod application fingers 2–4 (5.5 months), ALT thigh free flap with split thickness skin graft (5.5 months) ORIF left index metacarpal base (5.5 months prior)	6 months	ALT thigh flap debulking, Reconstruction of webspaces for index, long and ring fingers with z-plasties, Bilateral PL harvest, ECRL and ECRB harvest for EDC for index, long, ring, and small finger reconstructions, Tendon rod application, Full-thickness skin graft to ring finger	L/50%	L/50%	62 min/250 mm Hg	<15 mL
3	Series of I&Ds/wound VAC placements, Reduction of elbow, ex-fix spanning the elbow and wrist, Primary closure of elbow arthrotomy, Anconeus muscle tendon reattachment, Split thickness skin graft to elbow (All 3 years)	2.6 years	Free right lateral arm flap, Tenolysis of EDC, Left index, long, ring, and small finger MCP joints mobilization, Tendon transfer from PL to EPL, Tendon transfer from FCR to EDC index, long, ring, and small fingers with ECRL and ECRB harvest for extension grafts, Tendon side transfer EDM to EDC ring	R/50%	R/50%, L/50%	104 minutes RUE/250 mm Hg 124 minutes LUE/250 mm Hg	<10 mL
4	Excisional I&D down to joint and bone level (2 days)	3 days	I&D, PIA pedicle flap, ECRB and ECRL harvest for reconstruction of EIP, EDC EDC index, long, ring, and small fingers, and EDM. Intraoperative angiogram using SPY machine ×2. Split-thickness skin graft to forearm (2 × 8 cm)	R/50%	R/50% L/50%	90 min/200 mmHg	<10 mL
5	Extensor tendon repair (>14 years), Steroid injections (2 months), DRUJ capsule repair (1 month)	5.2 weeks	ECRL and ECRB Harvest for reconstruction of EDC EDC long, ring, and small fingers, DRUJ prosthesis placement	R/50%	R/50%	118 min/250 mm Hg	<10 mL

Notes: L, Left, R, Right. LUE, Left Upper Extremity. RUE, Right Upper Extremity. ORIF, Open Reduction and Internal Fixation. ORPF, Open Reduction and Pin Fixation. I&D, Irrigation and Debridement. ALT, Anterolateral. ECRB, Extensor Carpi Radialis Brevis. ECRL, Extensor Carpi Radialis Longus. Min, Minutes. Mm Hg, Millimetre of mercury. mL, milliliters. MCP, Metacarpophalangeal. TFCC, Triangular fibrocartilage complex. EPL, Extensor Pollicis Longus. EDC, Extensor Digitorum Communis. EIP, Extensor Indicis Proprius. EDM, Extensor Digitorum Minimi. FCR, Flexor Carpi Radialis. PL, Palmaris Longus. DRUJ, Distal Radioulnar Joint. PIA, Posterior Interosseous Artery. MCP, Metacarpophalangeal.

**Table 4:** Functional outcomes.

**Patient**	**Mayo Wrist Score**		**Return to activity**	**Follow up**
	**6 months post-op**	**12 months post-op**		
1	55	70	Without difficulties at 6 months	20 months
2	25	60	Without difficulties at 6 months	27 months
3	65	80	Without difficulties at 6 months	26 months
4	60	80	Without difficulties at 12 months	18 months
5	55	70	Without difficulties at 6 months	14 months

Notes: Postop, postoperative.

### Complications

Complications after the operation were as follows: Patient 1 had irritating subcutaneous sutures that were removed without difficulty or consequences on function shortly after the 12-month mark. Patient 2 had metacarpophalangeal joint capsular releases at the long, ring, and small fingers 3-months after the 12-month mark that helped considerably with his range of motion. Afterward, he was still noted to have significant Proximal interPhalangeal (PIP) flexion contractures at the long (30°) and ring (45°) fingers. However, his extrinsic extensor tendons were functioning well, and he could make a fist that was suitable for his everyday activities. Patient 4 had mild painless keloid scarring develop at the incision sites. Patient 5 was noted to have slight loosening of the DRUJ prosthesis at the 14-month mark that eventually required revision 2 years later. There was minimal donor site morbidity and no ruptures of parent tendons following harvest.

## Discussion

The current literature is limited regarding harvesting free tendon autograft strips from ECRB and ECRL [21–24], and in this study we presented our experience using these tendons in several upper extremity soft tissue reconstructions**.** A recent study by Lo *et al*. [23] used partial ECRL tendon grafts to repair chronic extensor pollicis longus (EPL) tears with tendon retraction in 23 patients. The reported cohort means for the total active motion (93.2%), pinch strength (95.4%), and the quick disabilities of the arm, shoulder, and hand score (6.0) demonstrated satisfactory functional outcomes [23]. In addition, Nkaoui *et al.* [23] used an identical technique and also obtained comparable results. Although our study used tendons from both ECRB and ECRL, Lo *et al*. cited that they opted to only use ECRL in their technique to preserve the integrity of ECRB as the main wrist extensor [23, 25]. ECRB has been suggested to have a centralizing role in the wrist [12] and to produce more force than ECRL [26]. However, based on our experiences we did not observe this to be a clinically relevant concern.

ECRB and ECRL synergistically coordinate wrist extension to counteract the flexion moment in gripping motions [27] and provide the power behind dart throwing motions [28]. This coactivation, similar direction of pull, and location next to functionally redundant muscles help to explain the minimal clinical consequences in strength or function following partial harvest of ECRB, ECRL, or both [10, 11, 14–16, 29–32]. A study by Wang *et al*. [24] used a tendon–bone graft partially harvested from ECRB and the base of the third metacarpal to treat 28 tendinous mallet fingers with residual extension lag after failing conservative treatment. Their results demonstrated satisfactory patient outcomes without residual postoperative wrist pain at final follow up [24]. While we showed similar results, their harvested graft size was considerably shorter (1 to 1.5 cm long) [24] than those used in our tendon gap reconstructions (2.5–16 cm). The surgical technique we used generally allowed us to obtain up to four strips of 12–15 cm tendon autografts between both arms [9], but a cadaveric study showed that up to 20 cm can be harvested (as seen with Patient 2, gap: 16 cm) [33].

We also consistently harvested tendon from the radial aspect of tendons, as this contained more of the upper muscles fibers and was generally longer [22]. Although PL and plantaris tendons are commonly harvested, one cadaveric study found 60% of them to be length insufficient, with <50% actually being usable for grafting [8]. In comparison, ECRB and ECRL are reliably present and easily accessible tendons [33]. Patient 3 lost their ECRB due to a severe crushing type injury several years prior to surgery; however, it was found in the contralateral arm predictably. Otherwise, the location of ECRB and ECRL further allowed for reconstruction and harvest site coverage with the same wound dressings and splint in our extensor tendon repair cases. This consolidated post-operative wounds care for patients and helped to make them more comfortable while recovering.

Flexor carpi radialis (FCR) is also a dependable source of partial autograft tendon and is often used in trapeziometacarpal interposition arthroplasty procedures [2, 34]. These tendons are commonly used for this procedure [2], so we opted to avoid harvesting it when possible in order to reserve it for possible future needs. A rare but feared complication that has been associated with partial autograft harvest is rupture of the providing tendon [34]. Of note, there were no source tendon ruptures seen in any of our cases or among those reported in similar ECRB and ECRL studies [21–24]. In fact, some studies noted that 1-year post-operative re-exploration of the graft sites showed these tendons to be intact, thicker, and stronger [22, 23]. We were vigilant to avoid rupture by longitudinally splitting tendons exactly midline using a 26 gauge surgical wire to optimize control and quality of the glide path [9, 34]. Wires were also inserted distally within the more clearly defined sheath before passing it proximally [9]. This retained connective tissue as a barrier to minimize inflammation, adhesions, and morbidity [6, 34]. A tendon stripper can be used as well if it is preferred by the surgeon or if a 26 gauge surgical wire is not available.

As seen during harvesting, the ECRB and ECRL tendons originate proximally as broad and tendinous fibers before flattening out as they course through the forearm and inserting distally as oval-shaped [33, 35, 36]. This allowed for extensor tendon repairs with minimal friction and efficient, smooth transfers of power [37]. However, these tendons are also thicker than PL or plantaris [12, 33] and may cause friction induced adhesions alongside flexor tendons running through narrow tunnels in the hand [38]. Therefore, PL or plantaris may be better suited for Zone 2 flexor tendon or pulley reconstructions due to their thin, long, smooth, and uniform gliding-type surfaces [39]. We recognized PL as an ideal transfer to EPL in Patient 3, as its surface qualities allowed for minimal mechanical abrasion while traveling obliquely near the rough, post-traumatic bony prominences observed intraoperatively at Lister’s tubercle [40].

## Conclusion

Our retrospective case series of five patients in which ECRB and ECRL tendons provided alternative sources of free partial autografts with satisfactory functional outcomes. This provides additional support for their use in repairing tendon gaps and ligament deformities in the upper extremity. Postoperatively, all five patients demonstrated subjective and objective improvement in function with minimal donor site morbidity. This study is inherently limited by its retrospective review and small sample size. Additionally, each patient had unique traumatic injuries with differing levels of severity. This technique may also not be appropriate for some patients, as previously discussed [9]. Surgeons are therefore advised to take a thorough pre-operative history and physical exam with possible imaging to confirm options, all while being prepared to consider other sources as necessary [2]. Success is also dependent on the resources available to surgeons. More high-quality prospective studies are needed to better evaluate the clinical efficacy of this technique.

## Author contributions

All authors made significant and meaningful contributions to the data collection and writing process of the manuscript.

## Conflict of interest statement

The authors declare that there are no conflicts of interest to report.

## Funding

This research did not receive any specific grant from funding agencies in the public, commercial, or not-for-profit sectors.

## Patient Consent

Informed consent was waived by the BLINDED Institutional Review Board (Reference: BLINDED) due to our anonymization of identifiable health information prior to use in research.
